# Mutational analysis of *TSC1* and *TSC2* genes in Tuberous Sclerosis Complex patients from Greece

**DOI:** 10.1038/s41598-017-16988-w

**Published:** 2017-12-01

**Authors:** Socratis Avgeris, Florentia Fostira, Andromachi Vagena, Yiannis Ninios, Angeliki Delimitsou, Radek Vodicka, Radek Vrtel, Sotirios Youroukos, Dimitrios J. Stravopodis, Metaxia Vlassi, Aristotelis Astrinidis, Drakoulis Yannoukakos, Gerassimos E. Voutsinas

**Affiliations:** 1 Laboratory of Environmental Mutagenesis and Carcinogenesis, Institute of Biosciences and Applications, National Centre for Scientific Research “Demokritos”, Athens, Greece; 2 Molecular Diagnostics Laboratory, INRaSTES, National Centre for Scientific Research “Demokritos”, Athens, Greece; 3 University Hospital and Palacky University Olomouc, Olomouc, Czech Republic; 4 First Department of Pediatrics, Aghia Sophia Children’s Hospital, University of Athens, Athens, Greece; 5 Section of Cell Biology and Biophysics, Department of Biology, National and Kapodistrian University of Athens, Panepistimiopolis, Zografou, Athens, Greece; 6 Laboratory of Protein Structure and Molecular Modelling, Institute of Biosciences and Applications, National Centre for Scientific Research “Demokritos”, Athens, Greece; 7 Division of Nephrology, Department of Pediatrics, College of Medicine, University of Tennessee Health Science Center; Tuberous Sclerosis Complex Center of Excellence, Le Bonheur Children’s Hospital; and Children’s Foundation Research Institute, Memphis TN 38103, Tennessee USA

## Abstract

Tuberous sclerosis complex (TSC) is a rare autosomal dominant disorder causing benign tumors in the brain and other vital organs. The genes implicated in disease development are *TSC1* and *TSC2*. Here, we have performed mutational analysis followed by a genotype-phenotype correlation study based on the clinical characteristics of the affected individuals. Twenty unrelated probands or families from Greece have been analyzed, of whom 13 had definite TSC, whereas another 7 had a possible TSC diagnosis. Using direct sequencing, we have identified pathogenic mutations in 13 patients/families (6 in *TSC1* and 7 in *TSC2*), 5 of which were novel. The mutation identification rate for patients with definite TSC was 85%, but only 29% for the ones with a possible TSC diagnosis. Multiplex ligation-dependent probe amplification (MLPA) did not reveal any genomic rearrangements in *TSC1* and *TSC2* in the samples with no mutations identified. In general, TSC2 disease was more severe than TSC1, with more subependymal giant cell astrocytomas and angiomyolipomas, higher incidence of pharmacoresistant epileptic seizures, and more severe neuropsychiatric disorders. To our knowledge, this is the first comprehensive *TSC1* and *TSC2* mutational analysis carried out in TSC patients in Greece.

## Introduction

Tuberous sclerosis complex (TSC) is a tumour suppressor syndrome of neurocutaneous origin. It exhibits an autosomal dominant mode of inheritance with an incidence varying from 1/6,000 to 1/10,000 births. Two-thirds of the cases are sporadic^1^. The disorder causes benign hamartomatous tumour growths in multiple organ systems, primarily in the brain, eyes, heart, kidney, skin and lungs^2^. Tubers growing in the brain are the main factors contributing to high morbidity and mortality in TSC. Brain involvement is often associated with the presence of epilepsy and TAND (Tuberous sclerosis Associated Neuropsychiatric Disorders)^3^. Clinical diagnosis of TSC is based upon identification of agreed and published major and minor diagnostic criteria^4,5^.

Twο genes have been identified as responsible for causing TSC: *TSC1* located on chromosome 9q34 and *TSC2* on 16p13.3. The *TSC2* gene was identified first by positional cloning. It contains 41 exons distributed over a genomic area of approximately 40 kb, produces a 5.5 kb transcript, and codes for a 200 kD protein named tuberin^6^. The *TSC1* gene was found to extend over a genomic region of 55 kb. It contains 23 exons, two of which are untranslated, generating an 8.5 kb transcript, from which a 130 kD protein product called hamartin is deriving^7^. Hamartin and tuberin were shown to be stabilized with the help of TBC1D7 and act in a multimeric complex, which suppresses cell growth by inhibiting the activation of mTOR, a master kinase that regulates multiple processes, including translation, ribosome and macromolecule biogenesis, neoangiogenesis, and autophagy^8,9^. In cells lacking wild type hamartin or tuberin, mTOR is dysregulated, leading to abnormal differentiation and development, and generation of enlarged cells, like the ones seen in TSC lesions^10^.

To date, more than 500 unique *TSC1* pathogenic variants have been identified in at least 1,350 probands/families, whereas another 1,400 mutations have been detected in *TSC2*, in over 3,600 probands/families^1^. Approximately, 80% of individuals diagnosed as definite TSC sufferers have been found to harbour pathogenic variants in *TSC1* or *TSC2*. The majority of remaining patients were shown to be mosaics or bear deep intronic mutations, if not possessing genetic changes in promoter or untranslated regions of these genes^11^. In this paper, we report a mutational analysis of *TSC1* and *TSC2* genes in 20 probands/families from Greece, of which 13 had a definite and 7 a possible clinical diagnosis of TSC. Additionally, we have assessed the distribution and type of mutations, and tried to build genotype-phenotype correlations between and within *TSC1* and *TSC2* cases.

## Results and Discussion

### Patients’ characteristics

In this work, we present results from twenty unrelated probands from Greece that were referred to the laboratory of Environmental Mutagenesis and Carcinogenesis (Molecular Diagnosis of Genetic Diseases Project) for TSC genetic testing. Thirteen probands (65%) had a definite clinical diagnosis, whereas the rest (35%) presented with clinical findings meeting the criteria for possible TSC diagnosis. Clinical characteristics of affected individuals were identified by doctors of various medical specialties and were reported to us by Paediatric Neurologists or the families themselves, based on the medical records of the patients. Clinical diagnosis was based on the revised diagnostic criteria for TSC^4,5^.

The phenotypic data along with neurobehavioral features are presented in Table 1. Nevertheless, it should be stressed that various clinical characteristics tend to appear at different ages of the affected individuals. Therefore, the phenotype of a number of young or very young patients could change as they get older. Eighty five percent (11/13) of the patients with a definite TSC diagnosis had a *TSC1* (31%) or *TSC2* (54%) mutation identified, whereas in patients with possible TSC diagnosis, only *TSC1* mutations have been detected in 29% of the cases (2/7). The patients in which no mutations have been identified (35%) were excluded from phenotype/genotype analysis.

**Table 1:** Clinical characteristics of TSC patients from Greece in the present study.

Patient	Sex	Gene*	TSC diagnostic status	Neurological findings			Renal findings	Pulmonary features	Ophthalmologic features	Dermatological findings			TAND		
				CD	SEGA	Seizures	AML	LAM	MRH	AF	SP	HM	LD	MR	ASD
1	F	TSC2	Definite	—	+	+ +	+	—	+	+	—	+	+ +	+	+
2	M	TSC2	Definite	+	+	+ +	+	—	+	+	+	+	++	++	+
3	M	—	Definite	+	—	+	—	—	—	+	—	—	+	—	+
4	F	TSC1	Definite	+	—	+	+	—	—	+	+	+	++	—	+
5	F	TSC2	Definite	+	+	++	+	+	+	+	—	—	++	++	+
6	M	TSC1	Definite	+	—	+	—	—	—	+	—	+	+	—	+
7	M	TSC1	Possible	+	—	+	—	—	—	—	—	—	+	—	+
8	F	TSC2	Definite	+	+	+	—	—	—	+	—	+	++	++	+
9	M	TSC2	Definite	—	+	+	—	—	—	—	+	—	+	+	+
10	F	TSC1	Definite	—	+	++	—	—	—	+	—	—	++	++	+
11	F	TSC2	Definite	+	+	++	—	—	—	—	—	+	na	na	na
12	F	—	Possible	+	—	+	—	—	—	—	-	—	+	—	—
13	F	—	Possible	+	—	+	—	—	—	—	—	—	na	na	na
14	F	TSC1	Definite	+	—	—	+	—	—	+	—	—	—	—	—
15	F	—	Definite	—	—	—	+	—	—	+	—	—	+	—	—
16	M	—	Possible	+	—	+	—	—	—	-	—	—	na	—	—
17	F	—	Possible	—	—	—	+	—	—	—	—	—	na	—	—
18	M	—	Possible	+	—	+	—	—	—	—	—	—	—	—	—
19	M	TSC2	Definite	+	—	+	—	—	—	—	—	+	na	—	na
20	F	TSC1	Possible	+	—	+	na	—	na	na	na	na	na	na	na

CD: Cortical dysplasias; SEGA: Subependymal giant cell astrocytomas; AML: Renal angiomyolipomas; LAM: Lymphangioleiomyomatosis; MRH: Multiple retinal hamartomas; AF: Angiofibromas; SP: Shagreen patches; HM: Hypomelanotic macules; TAND: TSC-Associated Neuropsychiatric Disorders; LD: Learning disabilities; MR: Mental retardation; ASD: Autism spectrum disorder; na: Information not available.

^*^Based on molecular diagnosis in the present study.

### Identification and characterization of mutations

In the present study, we performed mutational analysis in the coding exons and intron/exon junctions of both *TSC1* and *TSC2* in a total of twenty patients/families. TSC mutation screening was carried out in the probands, but whenever possible, the presence of pathogenic sequence variants was investigated in parental DNAs and in other family members as well. In total, thirteen mutations have been identified (Table 2). Five out of the thirteen (38.5%) have never been reported elsewhere; with the exception of the pathogenic variant identified one family, all the rest were *de novo* mutations. The novel mutations presented in this work are c.1681_1700del20 (p.Ser561Glyfs*20) and c.2263 C > T (p.Gln755*) in *TSC1*; c.648 + 1 G > T, c.826delA (p.Met276CysFs*17) and c.4942 A > T (p.Ile1648Phe) in *TSC2*. No recurrent mutations were identified in either gene.

**Table 2:** T*SC1* and *TSC2* disease-causing mutations in TSC patients from Greece.

Patient	Gene	Exon	Variant	Codon change	Mutation type	Inheritance	LOVD ID	ClinVar ID
4	*TSC1*	8	c.737 G > A	p.Arg246Lys	Missense	De novo	TSC1_00043	49094
6		9	c.901 C > T	p.Gln301*	Nonsense	Familial	TSC1_00056	49119
10		15	c.1681_1700del20	p.Ser561Glyfs*20	Deletion	Familial	—	—
20		18	c.2249 G > A	p.Trp750*	Nonsense	De novo	TSC1_00228	48922
14		18	c.2263 C > T	p.Gln755*	Nonsense	Familial	—	—
7		21	c.2701_2702delAG	p.Arg901Alafs*2	Deletion	De novo	TSC1_00779	—
19	*TSC2*	6	c.648 + 1 G > T	—	Splice-site	De novo	—	—
1		8	c.826delA	p.Met276Cysfs*17	Deletion	De novo	—	—
2		19	c.2206_2210dup	p.Cys738Leufs*35	Insertion	De novo	TSC2_00443	49600
8		20	c.2221–2 A > G	—	Splice-site	De novo	TSC2_00446	49815
9		23	c.2713 C > T	p.Arg905Trp	Missense	De novo	TSC2_00110	12404
5		37	c.4942 A > T	p.Ile1648Phe	Missense	De novo	—	—
11		39	c.5160 + 5 G > T	—	Splice-site	Familial	TSC2_00651	49430

**Figure 1:**
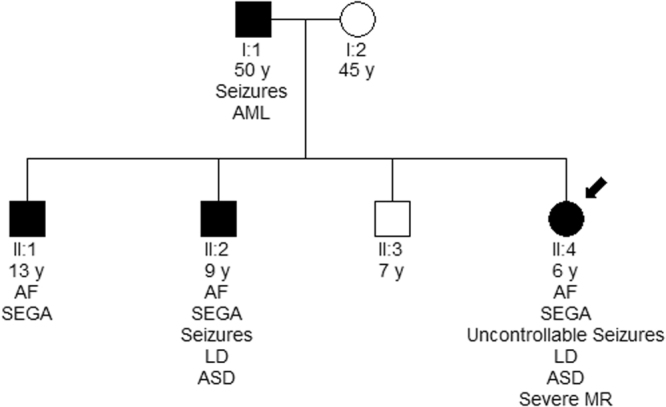
Family n° 10: Mutation c.1681_1700del20 leads to different clinical characteristics in the affected family members.

In more details, six disease-causing mutations were identified in *TSC1* (46%), and seven in *TSC2* (54%). *TSC1* sequence variants included 3 nonsense mutations producing premature termination codons; 2 deletions, which caused frameshifts also resulting in the truncation of the produced protein; and 1 missense mutation. Three of these were familial mutations, and another three were *de novo*, while four out of the six had been previously reported in LOVD (Leiden Open Variation Database, http://chromium.lovd.nl/LOVD2/TSC/home.php). In *TSC2*, the seven mutations detected consisted of 1 insertion and 1 deletion (frameshifts), 2 missense, and 3 splice-site mutations. Here, only one out of the seven mutations was of familial origin, with the rest being *de novo*. Four of those had been reported previously, whereas 3 were novel (Table 2). The mutations identified by Sanger sequencing were not clustered on a particular exon in either of the genes, whereas no Copy Number Variants were detected in *TSC1* or *TSC2* using MLPA analysis. Finally, only one out of the four TSC families identified here presented with multiple (>2) members affected by TSC1 disease. This family is of interest as the affected individuals present with significant phenotypic differences in clinical characteristics (Fig. 1), suggesting that additional factors interfere with development of the disease phenotype.

### Characterization of the additional variants identified

In this study, apart from the reported mutations with obvious or inferred pathogenic activity, additional variants have been identified. In total, 25 additional variants have been detected, from which 9 were found in *TSC1* and 16 in *TSC2*, whereas 4 among them have not been reported previously (1 at the *TSC1* locus and 3 at *TSC2*). These additional variants fell into two groups: (1) *TSC1* and *TSC2* variants co-existing with a TSC-causing mutation already detected in the bearers (Tables 3); and (2) *TSC1* and *TSC2* variants in patients with no TSC mutation identified (NMI) (Table 4).

**Table 3:** *TSC1* and *TSC2* additional variants in TSC patients from Greece with a pathogenic mutation identified.

Patient	Gene	Intron/Exon	Variant	Codon change	Variation Type	LOVD*				ClinVar**		Our data***
						I	II	III	IV	I	II	
14	*TSC1*	5′-UTR	c.1–7 C > T		5′-UTR	8	3	nk	nkp	—	—	1
8,9,15		exon 10	c.965 T > C	p.Met322Thr	Missense	114	12	+	nkp	13	b/lb	3
15		exon 12	c.1142–33 A > G		Intronic	12	—	+	nkp	3	b	1
15		exon 14	c.1335 A > G	p.Glu445Glu	Silent	119	9	+	nkp	9	b/lb	1
8,9,15		exon 15	c.1439–37 C > T		Intronic	12	—	+	nkp	2	b	3
9,15		exon 19	c.2392–35 T > C		Intronic	38	2	+	nkp	3	b	2
3		exon 22	c.2829 C > T	p.Ala943Ala	Silent	88	9	+	nkp	11	b/lb	1
7	*TSC2*	exon 10	c.1100 G > A	p.Arg367Gln	Missense	35	6	+	nkp	11	b/lb	1
5		exon 20	c.2221–99_2221–97del		Intronic	—	—	—	—	—	—	1
8		exon 38	c.4990–59 C > T		Intronic	—	—	—	—	—	—	1
1,11		exon 40	c.5202 T > C	p.Asp1734Asp	Silent	163	2	+	nkp	7	b/lb	2
15		exon 41	c.5313 G > C	p.Pro1771Pro	Silent	—	—	—	—	—	—	1

^*^Ι: Number of patients with the specific variant identified; II: Number of patients with a pathogenic variant identified; III: Found (+) in non-affected individuals, nk: not known; IV: Clinical significance suggested, nkp: no known pathogenicity.

**Ι: Number of independent submissions in the database; ΙΙ: Clinical significance suggested, b: benign, lb: likely benign.

***Number of times present in our database.

**Table 4:** *TSC1* and *TSC2* additional variants in TSC patients from Greece with no mutation identified (NMI).

Patient	Gene	Intron/Exon	Variant	Codon change	Variation Type	LOVD*				ClinVar**		Our data***
						I	II	III	IV	I	II	
12	*TSC1*	exon 15	c.1439–21delT		Intronic	—	—	—	—	—	—	1
17,18		exon 19	c.2502 + 51 A > G		Intronic	9	2	nk	nkp	1	np	2
13	*TSC2*	exon 10	c.976–63 G > A		Intronic	22	4	nk	nkp	1	np	1
13,16		exon 15	c.1600–14 C > T		Intronic	62	3	nk	nkp	6	b/lb	2
17		exon 22	c.2546–31 G > A		Intronic	1	1	nk	pnp	2	b	1
13,16		exon 22	c.2546–12 C > T		Intronic	18	3	nk	nkp	5	b	2
13,16		exon 22	c.2580 T > C	p.Phe860Phe	Silent	34	3	+	nkp	8	b/lb	2
13,16		exon 22	c.2639+44 C > G		Intronic	11	1	nk	nkp	2	b	2
12		exon 31	c.3815–15 G > A		Intronic	14	2	nk	nkp	4	b/lb	1
13,16		exon 40	c.5161–10 A > C		Intronic	91	2	+	nkp	5	b/lb	2
13		exon 41	c.5260–49 C > T		Intronic	25	9	nk	nkp	2	b	1
13		exon 41	c.5260–25 C > G		Intronic	5	1	nk	nkp	2	b	1
13,16		exon 41	c.5397 G > C	p.Ser1799Ser	Silent	91	12	+	nkp	6	b/lb	2

^*^Ι: Number of patients with the specific variant identified; II: Number of patients with a pathogenic variant identified; III: Found (+) in non-affected individuals, nk: not known; IV: Clinical significance suggested, nkp: no known pathogenicity, pnp: probably no pathogenicity.

**Ι: Number of independent submissions in the database; ΙΙ: Clinical significance suggested, b: benign, lb: likely benign, np: not provided.

***Number of times present in our database.

In total, 17 out of the 25 additional variants identified were intronic. Since these could possibly have pathogenic activity due to their possible involvement in alternative splicing, we have screened LOVD and ClinVar for all additional variants, and found that in a significant percentage they have been characterized as benign or likely benign (Tables 3 and 4). As a conclusion, it is likely that most of the additional variants identified in this work that are also present in LOVD and/or ClinVar, are polymorphisms with no apparent clinical significance.

### Genotype-Phenotype Correlations

#### Definite vs possible TSC cases

By definition, possible TSC patients present few symptoms, and very likely, a milder disease expression. Indeed, this was the case with our study, where among patients with possible TSC, displaying a generally milder disease phenotype, only 2 were found to bear a disease-causing mutation and this was detected in the *TSC1* gene.

#### TSC1 vs TSC2 disease

Generally, definite TSC2 patients presented with a more severe phenotype compared to definite TSC1 patients. While cortical dysplasias were often seen in both TSC1 and TSC2 disease (TSC1: 5/6 or 83%; TSC2: 5/7 or 71%), development of SEGAs was a characteristic of TSC2 probands only (TSC1: 1/5 or 20%; TSC2: 5/7 or 71%). Moreover, epileptic seizures were more severe in TSC2 patients, and this was followed by more pronounced TAND characteristics. Additionally, multiple retinal hamartomas and a single case of LAM were observed in TSC2 patients only, whereas AML were present in both TSC1 and TSC2 patients in rather similar percentages (TSC1: 2/6 or 33%; TSC2: 3/7 or 43%).

#### Familial vs de novo TSC mutations

A significantly higher percentage of patients with *de novo* TSC mutations (69%) were identified compared to familial cases (31%). Of the 4 familial mutations reported here, 3 (75%) were detected in *TSC1* and 1 (25%) in *TSC2*. In the three TSC1 families (6, 10 and 14), the pathogenic variant was passed on by the father. More specifically, all families suffered from premature termination of protein synthesis, with families 6 and 10 having one nonsense mutation each, and family 10 bearing a deletion of 20 nucleotides, which causes a frameshift. It is interesting to note that each of the 4 affected individuals in the latter family displays a different set of clinical characteristics, ranging from fibroadenomas plus SEGA with no epilepsy to SEGA with uncontrollable seizures, severe mental retardation and autism, all induced by the same *TSC1* mutation (Fig. 1).

**Figure 2:**
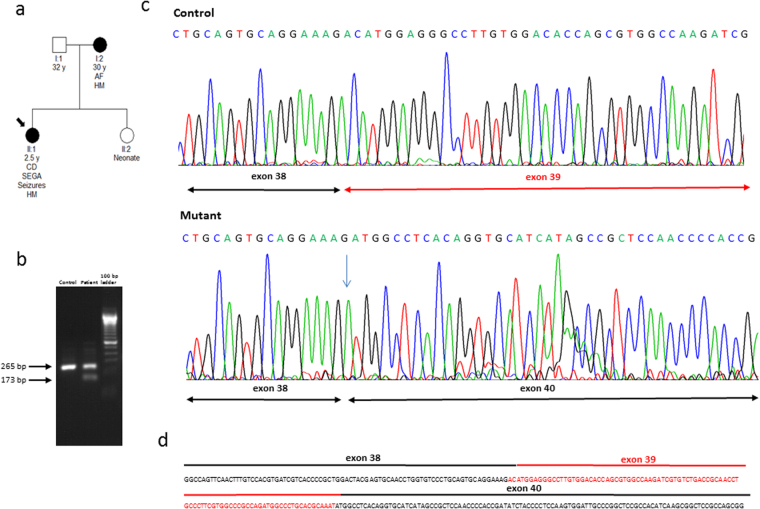
Family n° 11: RT-PCR analysis followed by Sanger sequencing. (**a**) Pedigree, (**b**) RT-PCR analysis, (**c**) Sanger sequencing of wild-type versus mutant cDNA, (**d**) wild-type sequence of TSC2 exons 38–40. Due to the c.5160+5 G > T splice-site mutation, exon 39 is absent from the mRNA produced by the mutant allele.

On the contrary, in TSC2 family 11, the mutation was transmitted by the mother, and it was a splice-site mutation. In this family, in order to determine whether the c.5160 + 5 G > Τ variant in intron 39 could be the cause of a splicing error, RT-PCR was performed on peripheral blood lymphocyte RNA obtained from the proband. Sequencing of the RT-PCR product revealed that this single nucleotide substitution was enough to induce skipping of *TSC2* exon 39 (Fig. 2).

Among the 9 *de novo* cases, 3 *TSC1* (33%) and 6 *TSC2* (67%) mutations were found. In total, we have identified 4 frameshift, 3 nonsense, 3 missense and 3 splice-site mutations.

### Prediction of structural consequences of TSC1 and TSC2 missense mutations

In order to assess possible pathogenicity of the Ile1648Phe (I1648F) *TSC2* missense variant, which is presented in this work for the first time, we compared 3D-models of Ile1648Phe *TSC2* variant versus wild-type, but also of p.Arg246Lys (R246K) *TSC1* missense variant versus wild-type, as an evaluation of our prediction.

**Figure 3:**
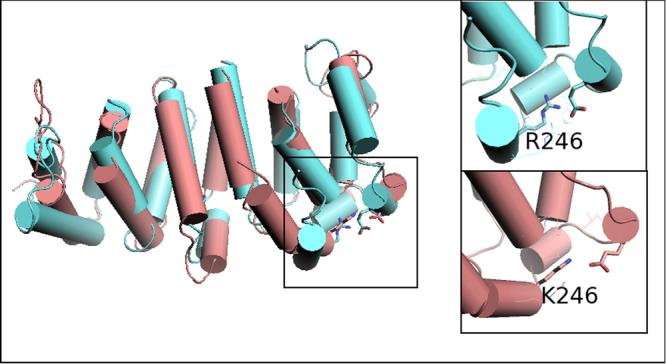
Prediction of possible structural consequences of TSC1 R246K missense mutation: Superposition of the final 3D-models (energy minimized average MD models) of the core domain (aa: 1–262) of wt hTSC1 and of its variant, R246K. The region of the mutation is boxed and magnified (right panel) for each one of the models, for reasons of clarity.

To investigate the effect of the TSC1 p.Arg246Lys missense mutation at the protein level, we first produced 3D-models of the core domain of wt hTSC1 protein and of its R246K variant, as described in Material & Methods. As shown in Fig. 3, the 3D-model of the TSC1 R246K variant is very similar to that of the wt protein. In addition, the 246 Arg/Lys side chains are involved in intra-molecular interactions in both models (Fig. 3). It is therefore unlikely that this sequence change affects either the structure or the interactions of the TSC1 protein. These observations are in line with data in the literature which show that the Arg246Lys substitution does not affect TSC1 function, and suggest that the effect of the TSC1 p.Arg246Lys mutation is rather a result of alternative splicing^12^.

**Figure 4:**
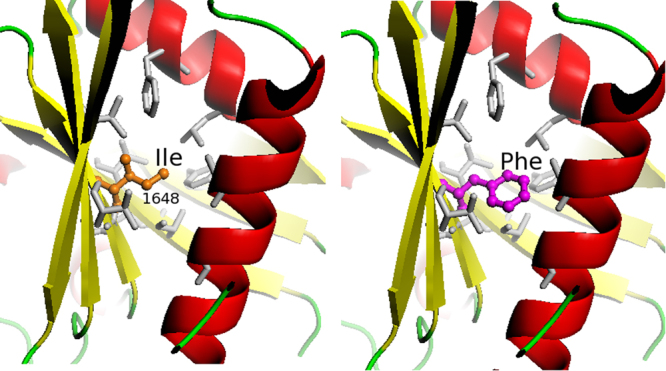
Prediction of possible structural consequences of TSC2 I1648F missense mutation: Details of the 3D-model of the catalytic domain of TSC2 at the Ile 1648 region for the wt (left panel) and the I1648F variant (right panel). The protein is depicted as ribbon model, including α-helices and β-strands. The amino acid 1648 is shown in ball-and-sticks and hydrophobic residues of the region are depicted as grey sticks.

On the contrary, as shown by mapping of the Ile1648Phe change on the 3D-model of the catalytic domain of TSC2 produced as described in Materials & Methods section (Fig. 4), substitution of the Ile residue at position 1648 by the much bulkier Phe residue, is anticipated to disrupt the structural integrity of this TSC2 domain due to steric hindrance with hydrophobic residues of the region (shown in grey sticks in Fig. 4). Therefore, it is most likely that the TSC2 p.Ile1648Phe mutation, by disrupting the structure of the catalytic domain of the TSC2 protein, impedes its GAP activity.

### Probands/families with no mutation identified (NMI)

In this work, in 7/20 probands/families, pathogenic mutations could not be identified. All 7 cases were *de novo*, with relatively mild symptoms of the disease. Nevertheless, since NMI refers to definite TSC patients^11^ and given the fact that here only 2 out of the 7 were definite TSC cases, with the remaining 5 being characterized as possible TSC, only these 2 are clear NMI, whereas the rest could be alternatively suffering from a disease other than TSC.

Generally, in NMI cases, pathogenic mutations could have been missed mainly because (a) they are found in genomic areas of *TSC1* or *TSC2* that are not covered during genetic analysis; or (b) the individuals are mosaics with just a small percentage of cells with a mutated *TSC1* or *TSC2*.

In studies similar to the present one, where genetic testing is carried out in a diagnostic setting, usually, analysis of promoter regions, 5′- and 3′-UTRs and deep intronic areas of *TSC1* and *TSC2* genes is not included^13–16^. In a few studies, where *TSC1* and *TSC2* promoter region analysis has been performed, the levels of mutations detected were either very low or null^17,18^.

The main reasons for exclusion of the above mentioned genomic areas in usual TSC genetic analysis are the limitations imposed by direct Sanger sequencing, but also the fact that in the majority of the patients, pathogenic mutations are detected in the exons and the intron/exon boundaries of the TSC genes^14,19,20^.

Nevertheless, nowadays, there is a trend towards the use of NGS-based technologies in TSC genetic analysis, exactly due to the fact that these new methods have the ability to cover readings of the whole length of *TSC1* and *TSC2* genomic areas, including the promoters, UTRs, and whole intron sequences, but also because of their sensitivity, where mutations can be detected in the presence of even only 9% of the minor allele^11,17,21^. Of course, on the other hand, the use of NGS-based genetic analysis cannot solve the problem of the variants of unknown clinical significance (VUS) and eliminate the need for functional analysis^11,17,21^.

Although the major contributions on TSC mutation scanning have been based on Sanger sequencing and less than 10 NGS papers have appeared in the TSC literature until this day, one cannot ignore the advantages of NGS in TSC genetic analysis over Sanger sequencing, which is posing some inherent limitations in our study.

## Conclusions

In this study, TSC1 and TSC2 disease percentages were rather similar (46% vs 54%), but detection of mutations proved more effective in patients having definite TSC (85%) than in patients having a possible TSC diagnosis (29%). Five new mutations were identified, while TSC1 disease presented with a milder phenotype, consistent with previous reports^14,19,20^. Most TSC2 mutations identified were *de novo* (86%). This was probably due to the more severe TSC2 disease phenotype, which likely prevents these individuals from having a family. In agreement with the above, most familial cases (75%) had TSC1 disease, likely due to its milder phenotype. The same was observed for all the patients with possible TSC diagnosis (100%), and the single family with multiple affected members. Nevertheless, familial cases could be slightly underrepresented in our study, since in some families only one of the parents was available for genetic testing. Finally, because TSC is one of the few rare diseases for which a targeted drug therapy is available, a more accurate genetic testing protocol should be introduced in order to help uncover the underlying molecular events in NMI individuals. A closer collaboration of scientists with TSC patient groups worldwide will probably shed more light on genotype/phenotype correlations in the near future, in the direction of improving the quality of life of patients suffering from TSC and families.

## Patients and Methods

### Patients

The study protocol and the informed consent forms were approved by the Bioethics Committee of NCSR “Demokritos”. All patients referred to the laboratory of Environmental Mutagenesis and Carcinogenesis, Molecular Diagnosis of Genetic Diseases Project, for genetic testing. In four cases (families 7, 8, 9 and 11) prenatal diagnosis has also been performed. Before molecular diagnosis, written informed consent forms were signed by all probands or parents, whereas after analysis, families were informed in detail on the outcome of the genetic test. Finally, the study was in agreement with the 1975 Helsinki statement, revised in 1983.

### Mutation detection

Genomic DNA was extracted from peripheral blood lymphocytes according to the standard saturated salt-chloroform extraction protocol. Purity and concentration of isolated DNA were measured using a NanoDrop™ spectrophotometer, while the quality of genomic DNA was evaluated through agarose gel electrophoresis. The entire translated regions of *TSC1* (exons 3 to 23) and *TSC2* (exons 1 to 41) were PCR-amplified and then directly sequenced using the Sanger method. All primer sequences and PCR conditions used are shown in Tables S1 and S2 (see Supplemental Materials and Methods). Cycle sequencing reactions were performed using the v3.1 BigDye Terminator Cycle Sequencing kit (Applied Biosystems, Foster City, CA), and then analysed on an ABI Prism® Genetic Analyzer. Sequences obtained were aligned against reference sequences from the Genbank (Accession Numbers: NG_012386.1 (*TSC1*) and NG_005895.1 (*TSC2*)), and examined for the presence of variants. Family members of mutation carriers were being informed in counselling sessions, and if they consented, they were subjected to genetic analysis for the specific mutation. The origin of mutations (inherited or *de novo*) was inferred after testing both parents (when available). No paternity test was performed.

### Confirmation of the presence of an aberrant mRNA splice variant by RT-PCR analysis followed by DNA sequencing

In order to test for possible splicing anomalies within *TSC2* mRNA transcripts (family 11), we performed total RNA extraction from peripheral blood lymphocytes, using ΤRI REAGENT (Molecular Research Center Inc, Cincinnati, OH). Subsequent cDNA synthesis was carried out using M-MLV RT (Life Technologies, Carlsbad, CA). In the particular family, RT-PCR analysis was performed with the help of a forward primer on exon 38 and a reverse primer on exon 41. RT-PCR products were sequenced and analysed on an ABI Prism® Genetic Analyzer.

### Multiplex ligation-dependent probe amplification (MLPA)

Possible large rearrangements in *TSC1* and *TSC2* were assessed by Multiplex Ligation-dependent Probe Amplification (MLPA) using the SALSA MLPA P124 and SALSA MLPA P046, respectively, following manufacturer’s instructions (MRC-Holland, Netherlands).

### Molecular Modeling of missense mutations

The 3D-model of the core domain of human TSC1 (aa: 1–262) was obtained from the Swiss_Model^22^ and was based on the known crystal structure of the TSC1 core domain from *S. pombe* ^23^ (PDB entry: 1KK0). The initial models of both the wild-type (wt) hTSC1 core domain and of its R1246K variant were subsequently subjected to molecular dynamics (MD) simulations in explicit water, using a procedure similar to that applied in Voukkalis *et al*. 2016^24^. Three independent, 50 ns long MD simulations were performed for each molecule. The 3D-model of the GAP domain of human TSC2 (aa: 1502–1756) was constructed using a combination of the Swiss-Pdb Viewer program^25^ and the Phyre server^26^. The known crystal structure of the Rap1GAP catalytic domain^27^ (PDB entry: 1SRQ) was used as template, for this purpose.

### Data availability

Data are available upon request.

## Electronic supplementary material

Tables S1 and S2
